# Venous Thromboembolism as an Adverse Effect During Treatment With Olanzapine: A Case Series

**DOI:** 10.3389/fpsyt.2019.00330

**Published:** 2019-05-15

**Authors:** Jiri Masopust, Vera Bazantova, Kamil Kuca, Blanka Klimova, Martin Valis

**Affiliations:** ^1^Department of Psychiatry, Charles University in Prague, Faculty of Medicine in Hradec Kralove and University Hospital, Hradec Kralove, Czechia; ^2^Department of Neurology, Charles University in Prague, Faculty of Medicine in Hradec Kralove and University Hospital, Hradec Kralove, Czechia; ^3^Department of Chemistry, Faculty of Science, University of Hradec Kralove, Hradec Kralove, Czechia; ^4^Biomedical Research Center, University Hospital Hradec Kralove, Hradec Kralove, Czechia

**Keywords:** antipsychotics, olanzapine, venous thromboembolism, side effects, risk factors

## Abstract

**Objective:** Venous thromboembolism (VTE) is a serious multifactorial disorder. Patients with severe mental illness have a higher risk of developing the condition compared to the general population.

**Methods:** We observed 10 cases of VTE in patients with mental illness who were treated with the antipsychotic drug olanzapine. The diagnosis of VTE was made at the University Hospital Hradec Kralove (UH HK) from 2004 to 2013. VTE was objectively determined by imaging techniques (duplex ultrasonography, CT angiography) and laboratory tests (D-dimer). The average age was 46 years. The clinical manifestation of VTE was deep vein thrombosis in nine cases, including one case of simultaneous pulmonary embolism and one case of a concurrent ischemic cerebrovascular accident (iCVA). None of our patients had a history of malignant disease, trauma, or surgery.

**Results:** Apart from antipsychotic medication, all the patients had clinical or laboratory risk factors for VTE. The most frequent clinical risk factors were obesity (n = 7) and smoking (n = 6). The most frequent laboratory risk factors were increased levels of FVIII (n = 4), mild hyperhomocysteinemia (n = 3), and factor V Leiden mutation (n = 2). VTE developed within 3 months after antipsychotic drug initiation in three patients and within 6 months in three patients.

**Conclusion:** Olanzapine can be considered a precipitating factor for VTE formation. When olanzapine is administered, we need to monitor for clinical signs and symptoms of VTE, especially when other risk factors are present.

## Introduction

Venous thromboembolism (VTE) is a serious and potentially life-threatening condition. Manifestations of VTE include deep vein thrombosis (DVT) and pulmonary embolism (PE). VTE is associated with high morbidity and mortality. Considerable amounts of money are also spent on the treatment of VTE in the developed countries of Europe and North America. VTE affects approximately 1.0–1.8 people per 1,000 annually (1, 2). The incidence rises exponentially with age, and the risk significantly increases in people over the age of 40 years (3). Multiple risk factors are involved in the development of VTE. VTE risk factors can be divided into physiological and pathophysiological, clinical and laboratory, and congenital and acquired factors (**Table 1**) (1, 2, 4).

**Table 1:** Risk factors for VTE [adapted from Refs. (1, 2, 4)].

Congenital	Antithrombin deficiency
	Protein C deficiency
	Protein S deficiency
	Factor V Leiden mutation
	Factor II mutation
Acquired	Increasing age
	Malignancy
	Antiphospholipid syndrome
	Inflammatory bowel disease
	Systemic lupus erythematosus
	Nephrotic syndrome
	Overweight and obesity
	Microalbuminuria
External	Infectious disease
	Surgery, trauma, immobilization
	Pregnancy and puerperium
	Oral contraceptives
	Hormonal replacement therapy
	Antipsychotic drugs
	Air transport (long haul)
Mixed	High factor VIII levels
	APC resistance
	Hyperhomocysteinemia
	High factor IX levels
	High factor XI levels
	Fibrinolysis abnormalities
Partially confirmed	High CRP levels
	Smoking
	Dyslipidemia
	Male sex

VTE, venous thromboembolism; CRP, C-Reactive Protein.

The congenital risk factors for VTE have varying clinical implications for the patient as they confer different degrees of relative risk for VTE development. The risk of thrombosis rises significantly when multiple factors occur together. In older patients, acquired risk factors occur more frequently, which explains the significantly increased incidence of VTE in the elderly population.

The pathophysiological mechanism underlying VTE formation is the development of a relative thrombophilic state. This involves a shift in the hemostatic system towards thrombosis. If a genetic disposition is prominent in the etiopathogenesis of VTE, patients are determined to have hereditary thrombophilia. If external risk factors prevail, the condition is determined to be an acquired hypercoagulable state. A hypercoagulable state may result from the presence of any of the conditions belonging to the so-called Virchow triad (venous stasis, hypercoagulability, and vessel wall damage) (1, 2).

In patients with a mental illness, the risk of VTE is increased for several reasons. In a previous study, we found significantly increased activation of markers of thrombogenesis (sP-selectin, D-dimer, FVIII) in untreated patients experiencing a first episode of psychosis compared to healthy volunteers (5). The markers of thrombogenesis continued to be elevated after 1 year of treatment (6). Antiphospholipid antibodies (APAs), especially anticardiolipin antibodies (ACLAs), may serve as a marker of autoimmune reactivity, or they may directly interfere with phospholipid metabolism in schizophrenia patients. They may also have prothrombogenic effects. Inflammatory markers are also elevated during acute psychosis, which may lead to an increased risk of pathological blood coagulation (7). Hyperhomocysteinemia, which is also a risk factor for VTE, can occur in patients with mental disorders who also smoke, have poor dietary habits, and consume excessive amounts of coffee

Antipsychotic medications are another risk factor for VTE in patients with a psychotic disease (8). Antipsychotic drugs contribute to the development of a hypercoagulable state through several potential mechanisms, including drug-induced sedation, obesity, hyperprolactinemia, increased platelet aggregation, and elevation of APA (5, 9).

According to a meta-analysis by Barbui et al. (10), exposure to antipsychotic treatment results in a 50% increased risk for VTE. In another meta-analysis, the low-potency antipsychotic agents had the highest odds of VTE (OR 2.91; 95% CI 1.81–4.71), followed by second-generation antipsychotics (OR 2.20; 95% CI 1.22–3.96) and then the traditional neuroleptics (OR 1.72; 95% CI 1.31–2.24) (11). The highest risk of VTE emerges during the first 3 months after initiation of antipsychotic treatment (12). Other studies found the highest risk in the first month after the initiation of an antipsychotic (13, 14). In addition to the time period following drug administration and the antipsychotic drug class used, other risk factors include using higher drug doses, using a combination of several antipsychotic drugs, and parenteral administration of antipsychotic agents (15).

Through a long-term project named ANTRE (ANTipsychotics, ThRombosis, Embolism) involving interdisciplinary cooperation within the University Hospital in Hradec Kralove, we have been aiming to develop a comprehensive approach to the issue of VTE in patients treated with antipsychotic drugs. Cases of VTE development are carefully recorded. In addition to isolated case reports of VTE development during treatment with different antipsychotic agents, we recorded 10 cases involving the administration of olanzapine.

## Patients and Methods

The patient group consisted of a total of 10 patients (women n = 3) who were treated with olanzapine. VTE occurrence was monitored prospectively between 2004 and 2013 in a specialized outpatient unit for VTE treatment. The average age of the patients was 46 years, with a range of 17 to 71 years. Three patients had a diagnosis of delusional disorder, two had paranoid schizophrenia, and two had bipolar disorder. The other patient diagnoses were an addiction to multiple narcotic drugs, behavioral disorder related to pregnancy and puerperium, and mental retardation with a behavioral disorder. The duration of psychiatric illness prior to the occurrence of VTE was determined. We also determined the duration and dose of olanzapine utilized prior to VTE and recorded other psychotropic drugs taken concomitantly. Information was collected from patient-reported history, the hospital information system, and reports by attending outpatient psychiatrists. None of the patients had previously suffered from VTE. Patient history was used to determine if there was a family history (FH) of VTE. In the patient group, VTE occurred either during hospitalization or during outpatient care. VTE diagnosis and subsequent treatment were conducted by an internist. If clinical symptoms suggested VTE, then additional examinations were conducted. In all cases, VTE was verified using imaging techniques (duplex ultrasonography, CT angiography) and laboratory methods (D-dimer). Blood tests were used to detect laboratory risk factors. All laboratory tests were conducted in the same laboratory.

Due to the case study nature of the data, we mostly used methods of descriptive statistics. Frequencies for sex, clinical and laboratory risk factors, smoking, and VTE together with averages for age, weight gain, dose of olanzapine, and length of use were calculated. A paired-sample t-test was used for weight gain.

## Results

VTE manifested clinically as DVT in the lower limbs in nine patients. In one of these patients, PE also developed at the same time. In one patient, VTE manifested clinically as neurological symptoms without symptoms of DVT. These atypical symptoms were due to a paradoxical embolization in the setting of a heart malformation, patent foramen ovale. Clinical or laboratory risk factors for VTE development were present in all the patients. The most frequent clinical risk factors were obesity (n = 7) and smoking (n = 6). The most frequent laboratory risk factors were elevated FVIII level (n = 4) and mild homocysteinemia (HCY) (n = 3). In these patients, mild HCY was always present together with elevated FVIII level. FV Leiden mutation was detected in two patients. Both clinical and laboratory risk factors were present in half (n = 5) of the patients. Positive FH for VTE was found in three patients. In one case, VTE resulted in the death of an affected family member. In six patients, VTE developed within 6 months of olanzapine initiation, and within 3 months in three patients. VTE developed during hospitalization in three cases. The average dose of olanzapine was 14.5 mg daily, and the average length of use was 14 months prior to VTE. Only one patient was using olanzapine monotherapy. There was also statistically significant weight gain recorded during treatment with olanzapine (p = 0.031). The results are summarized in **Table 2**.

**Table 2:** Characteristics of the patient group, including demography, diagnosis, duration of illness, dose and time of olanzapine use, adjuvant medication, and BMI before olanzapine initiation and during VTE development.

	Sex (age range)	Diagnosis (years of illness)	Psychotropic drugs		BMI before/after OLA treatment	VTE risk factors		VTE		
			OLA in mg (months of use)	Other (mg)		clinical	laboratory	PH	FH	Therapy
**1**	Male (51–55)	F22 (24)	5 (2)	alprazolam 1	26.4/27.6	−	mild HCY elevated FVIII	DVT PE	−	LMWH warfarin
**2**	Male (51–55)	F22 (0.5)	10 (6)	sertraline 100	23.7/23.7	smoking	FII mutation (het)	DVT	+	LMWH warfarin
**3**	Male (36–40)	F20 (10)	10 (6)	zotepine 100 levomepromazine 25	30/34	obesity smoking	FV Leiden mutation (het)	DVT	−	LMWH warfarin alteplase SCF
**4**	Female (51–55)	F22 (32)	20 (17)	citalopram 40 oxazepam 15	29.7/30.9	obesity smoking immobilization	mild HCY elevated FVIII	DVT	−	LMWH warfarin
**5**	Female (71–75)	F31 (19)	20 (6)	escitalopram 10 trazodone 150 oxazepam 15 lamotrigine 25	29.8/30.8	obesity LE varicose veins immobilization	−	DVT	+	LMWH warfarin
**6**	Male (56–60)	F19.2 (10)	10 (12)	clonazepam 5 promethazine 50	30.9/30	obesity smoking	−	DVT	−	LMWH warfarin
**7**	Female (31–35)	F53.1 (0.2)	20 (0.17)	venlafaxine 225	28.2/32.6	obesity immobilization puerperium	−	DVT	+	LMWH warfarin
**8**	Male (36–40)	F20 (13)	30 (84)	clonazepam 1	28/28.6	smoking	elevated FVIII	DVT	−	warfarin
**9**	Male (15–19)	F72.1 (17)	10 (1)	levomepromazine 125 chlorprothixene 250	25.2/31.7	obesity	FV Leiden mutation (het) elevated FVIII mild HCY	DVT	−	LMWH warfarin
**10**	Male (41–45)	F30.2 (0.7)	10 (3.5)	−	31.7/30.5	obesity smoking LE varicose veins	−	paradoxical embolization	−	ac. acetylsalicylicum occluder implantation

OLA, olanzapine; BMI, body mass index; VTE, venous thromboembolism; DVT, deep vein thrombosis; PE, pulmonary embolism; iCVA, ischemic cerebrovascular accident; LE, lower extremities; HCY, homocysteinemia; het, heterozygote; PH, personal history; FH, family history; LWMH, low-molecular-weight heparin; SCF, subrenal cava filter.

## Discussion

Olanzapine is a widely used, affordable, and effective second-generation antipsychotic drug. It is indicated for the treatment of schizophrenia and bipolar disorder. In clinical practice, the spectrum of its use is much wider. Off-label indications include psychotic disorders of various etiologies (toxic and organic), behavioral disorders in patients with dementia, eating disorders, and personality disorders. The relatively high incidence of olanzapine-related VTE may be distorted by the high number of patients treated with this antipsychotic agent.

A possible prothrombogenic mechanism of olanzapine at the molecular level is an affinity for 5-HT_2A_ serotonin receptors. Blocking these receptors results in increased platelet aggregation and increased blood coagulability (16). Blockade of α_1_ adrenergic receptors by olanzapine may cause hypotension and thereby venous stasis in the lower limbs. Another mechanism may be olanzapine-induced production of the antiphospholipid antibodies: lupus anticoagulant (LA) and ACLAs (17). Increasing these APA titers is associated with a prothrombogenic state (18). Olanzapine may also induce a temporary increase in prolactin levels early in the course of treatment. Hyperprolactinemia correlates with increased levels of P-selectin, a platelet activation marker (19).

Metabolic symptoms caused by olanzapine represent an indirect mechanism for VTE development. The prothrombogenic metabolic symptoms that often occur during olanzapine treatment include hyperglycemia, hyperleptinemia, dyslipidemia, and weight gain (20). Weight gain is mainly due to the blockade of H_1_ histamine and 5-HT_2C_ serotonin receptors by olanzapine.

Obesity [body mass index (BMI) ≥ 30)] is associated with an up to two-fold increase in the risk of VTE development. Obese patients have reduced fibrinolytic activity and capacity. Higher levels of the procoagulation factors FVIII and FIX also occur (21). Central obesity is also accompanied by a procoagulant and hypofibrinolytic state related to inflammation, oxidative stress, dyslipidemia, and ectopic fat deposition (22). In our group, obesity was identified in seven patients. The patients’ body weight increased significantly during olanzapine treatment.

Another indirect risk factor associated with olanzapine is sedation, which reduces physical activity, leading to a reduction in blood flow. Venous stasis, which is one of the factors from the Virchow triad, increases the risk of VTE. The level of sedation in our patients could also have been potentiated by adjuvant medication. In 50% (n = 5), benzodiazepines were administered together with olanzapine. In 20% (n = 2), the sedating antipsychotic agent levomepromazine was used with olanzapine.

The risk of VTE development increases significantly during prolonged hospitalization. Patients with a mental disorder require hospitalization frequently and repeatedly. In our group, VTE occurred during hospitalization in three patients. During hospitalization, a general reduction in physical activity occurs, and sedatives are often administered at higher doses compared to outpatient treatment. Some patients also require physical restraint. During physical restraint, such as strapping the patient to a bed, injury or compression of a limb may occur. Venostasis and vascular endothelium damage can also occur, which increases the risk for VTE (23). In our group, physical restraint was used in only one patient.

Literature reports that the risk of VTE is highest in the first 3 months after initiation of antipsychotic treatment. This finding was confirmed in 30% of patients in our group; however, in 60%, VTE developed within 6 months after olanzapine initiation. Our findings confirm those of other studies, which found that the highest risk of VTE development exists in the early stages of antipsychotic treatment (24).

FV Leiden mutation is the most frequently occurring laboratory risk factor for VTE development in the general population (1). It can be detected in 20–25% of patients with a diagnosis of VTE. This corresponds with the findings from our group, in which FV Leiden mutation was identified in 20% (n = 2) of the cases.

This is the largest group of patients with VTE occurrence during olanzapine treatment ever described in the literature. The merits of our work include meticulous VTE diagnosis using modern imaging methods and laboratory testing. The number of VTE cases reported during treatment with olanzapine may be influenced by the fact that it is a very frequently used antipsychotic drug.

Antipsychotic agents, in general, may be regarded as one risk factor for VTE development. Patients with psychosis have a higher incidence of cardiovascular risk factors (obesity, diabetes mellitus, arterial hypertension, dyslipidemia, and smoking) that are also associated with a higher risk for VTE development (25). In VTE pathogenesis, it is difficult to differentiate between the role of the underlying psychiatric condition, hospitalization or physical restraint, and the action of antipsychotic drugs. The precise biological mechanisms of increased VTE during antipsychotic use remain unclear.

When selecting particular treatment, the presence of VTE risk factors should be considered for every patient. Treatments should be selected only if they do not further increase the risk. During antipsychotic therapy, the risk for VTE development needs to be monitored, and prophylactic measures should be initiated in a timely manner. Diagnosing VTE in mentally ill patients may be more difficult than in the general population. High-risk patients treated with antipsychotic drugs should receive clear information on the possible adverse effects of the treatment, including clinical signs and symptoms of venous thrombosis and PE, and on the necessity of seeking medical attention if such symptoms occur.

In hospitalized immobilized psychiatric patients, the degree of VTE risk needs to be determined so that preventive measures can be taken (26). Whenever clinical symptoms of VTE occur, the diagnosis needs to be confirmed or ruled out immediately by imaging and laboratory tests.

Further prospective studies are needed to shed more light on the relationship between VTE and antipsychotic agents, focusing on risk quantification for the individual antipsychotic drugs, and to elucidate the basic biological mechanisms.

## Conclusion

We have described 10 patients with VTE occurrence during treatment with olanzapine. In all the cases, additional clinical or laboratory risk factors were detected. Olanzapine can be considered a precipitating factor for VTE development. During olanzapine administration, patients need to be monitored for clinical signs of VTE, particularly when multiple risk factors are present.

## Data Availability Statement

The data sets generated and/or analyzed during the current study are available from the authors.

## Ethics Statement

This study was approved by the ethical committee of FNHK (Hradec Kralove, Czechia). Informed consent was obtained from all individual participants included in the study.

## Author Contributions

MV, JM, and VB contributed to the data collection. MV, JM, BK, and KK contributed to the preparation of the draft.

## Funding

This paper was supported by the research project PROGRES Q40 at the Medical Faculty of Charles University and by MH CZ–DRO (UHHK 00179906). It was also supported by the long-term development plan of UHK.

## Conflict of Interest Statement

The authors declare that the research was conducted in the absence of any commercial or financial relationships that could be construed as a potential conflict of interest.
